# The *Staphylococcus aureus* regulatory program in a human skin-like environment

**DOI:** 10.1128/mbio.00453-24

**Published:** 2024-03-28

**Authors:** Flavia G. Costa, Krista B. Mills, Heidi A. Crosby, Alexander R. Horswill

**Affiliations:** 1 Department of Immunology and Microbiology, University of Colorado Anschutz Medical Campus, Aurora, Colorado, USA; 2 Department of Veterans Affairs, Eastern Colorado Healthcare System, Aurora, Colorado, USA; The University of Texas Health Science Center at Houston, Houston, Texas, USA

**Keywords:** *Staphylcoccus aureus*, *Staphylococcus*, skin colonization, skin-like media, RNA-seq, transcriptional regulation, adherence

## Abstract

*Staphylococcus aureus* is a Gram-positive pathogen responsible for the majority of skin and soft tissue infections (SSTIs). *S. aureus* colonizes the anterior nares of approximately 20%–30% of the population and transiently colonizes the skin, thereby increasing the risk of developing SSTIs and more serious infections. Current laboratory models that mimic the skin surface environment are expensive, require substantial infrastructure, and limit the scope of bacterial physiology studies under human skin conditions. To overcome these limitations, we developed a cost-effective, open-source, chemically defined media recipe termed skin-like medium (SLM) that incorporates key aspects of the human skin surface environment and supports growth of several staphylococcal species. We utilized SLM to investigate the transcriptional response of methicillin-resistant *Staphylococcus aureus* (MRSA) following growth in SLM compared to a commonly used laboratory media. Through RNA-seq analysis, we observed the upregulation of several virulence factors, including genes encoding functions involved in adhesion, proteolysis, and cytotoxicity. To further explore these findings, we conducted quantitative reverse transcription-PCR (qRT-PCR) experiments to determine the influence of media composition, pH, and temperature on the transcriptional response of key factors involved in adhesion and virulence. We also demonstrated that MRSA primed in SLM adhered better to human corneocytes and demonstrated adhesin-specific phenotypes that previously required genetic manipulation. This improved adherence to corneocytes was dependent on both acidic pH and growth in SLM. These results support the potential utility of SLM as an *in vitro* model for assessing staphylococcal physiology and metabolism on human skin.

**IMPORTANCE:**

*Staphylococcus aureus* is the major cause of skin diseases, and its increased prevalence in skin colonization and infections present a need to understand its physiology in this environment. The work presented here outlines *S. aureus* upregulation of colonization and virulence factors using a newly developed medium that strives to replicate the human skin surface environment and demonstrates roles for adhesins clumping factor A (ClfA), serine-rich repeat glycoprotein adhesin (SraP), and the fibronectin binding proteins (Fnbps) in human corneocyte adherence.

## INTRODUCTION

The skin is the largest organ in the human body (1). This organ is highly stratified and interspersed with skin appendages including hair, sebaceous glands, eccrine glands, and apocrine glands (2). The stratum corneum is the outermost layer of the skin and is composed of 15–25 layers of nonviable, anucleate corneocytes that are assembled in a “brick-and-mortar” arrangement that prevents water loss and protects the body against environmental challenges and pathogens (2). Over the course of human evolution, genes encoding functions of the human skin have acquired several mutations that resulted in a unique skin surface environment encountered by commensals and pathogens alike (3). For example, the ubiquitously distributed eccrine glands produce sweat at up to 0.5–3.5 L/h combined and are a unique attribute of human skin physiology (3–5). Additionally, the human skin surface is more acidic (pH 4.1–5.8) than those of other mammals commonly used in skin infection models (6, 7).

*Staphylococcus aureus* is the most common pathogen isolated from skin and soft tissue infections (8). Treatment and prevention of *S. aureus* skin infections are further complicated by the prevalence and transmission of community-acquired, methicillin-resistant *S. aureus* (MRSA) strains (8–10). The CA-MRSA USA300 strain, in particular, has become epidemic and drives the increased prevalence of skin and soft tissue infections in the last two decades (10). MRSA infection risk is correlated to nasal carriage, a reservoir from which MRSA can transiently colonize the skin (11).

The broad host and site range of *S. aureus* is reflected in its metabolic and physiological adaptability to these environments (12, 13). *S. aureus* is a facultative anaerobe that can use nitrate as a terminal electron acceptor, with a preference for oxygen (14). The glycolysis, tricarboxylic acid (TCA) cycle, and pentose phosphate pathways are complete in *S. aureus* and *Staphylococcus epidermidis*, providing the precursors required for all macromolecules (13). Despite this, isolates of *S. aureus* have been characterized with diverse amino acid auxotrophies, the most common of which include arginine, proline, cysteine, and valine (13, 15–17). Additionally, staphylococci require supplementation of nicotinic acid and thiamin, and some coagulase-negative isolates also require supplementation of biotin and/or pantothenate (18, 19).

In the host, *S. aureus* secretes extracellular proteases and toxins, cell surface adhesins, and other factors that facilitate immune evasion, nutrient acquisition, and dissemination (20). Our understanding of staphylococcal physiology and metabolism has predominantly been studied in the context of infection, and studies of staphylococcal physiology on human skin in the early stages of colonization preceding infection have been restricted by cost and other limitations of currently used laboratory models (7, 21). To close this gap, we developed a skin-like medium (SLM) for the study of staphylococci that incorporates metabolites derived from eccrine sweat and stratum corneum turnover. We also incorporated the acidity, temperature, and buffering capacity of a healthy skin barrier into the media recipe and growth conditions. This media promoted growth of seven different staphylococcal species isolated from both infectious and colonization contexts. To inquire how *S. aureus* responds to these conditions, we analyzed transcriptional changes of the CA-MRSA USA300 strain LAC* following growth in SLM in comparison to a commonly used growth medium, trypto soy broth (TSB). These transcriptional changes were compared to quantitative reverse transcription PCR (qRT-PCR) data from previously published *ex vivo* human skin explant experiments as a comparator. Differentially regulated genes encoding key colonization and virulence factors were validated by qRT-PCR, and the influence of temperature and pH on the transcription of these loci was assessed. The utility of SLM was demonstrated with assessment of MRSA adhesion to human corneocytes, an assay that previously required regulatory manipulation of adhesins to assess their role in this key aspect of skin colonization. The results support that growth in SLM stimulates a dramatic transcriptional response in *S. aureus*, including the expression of key virulence factors, and can expand the applicability of host-microbe studies performed at the research bench.

## RESULTS

### Considerations and development of the SLM

**Fig 1:**
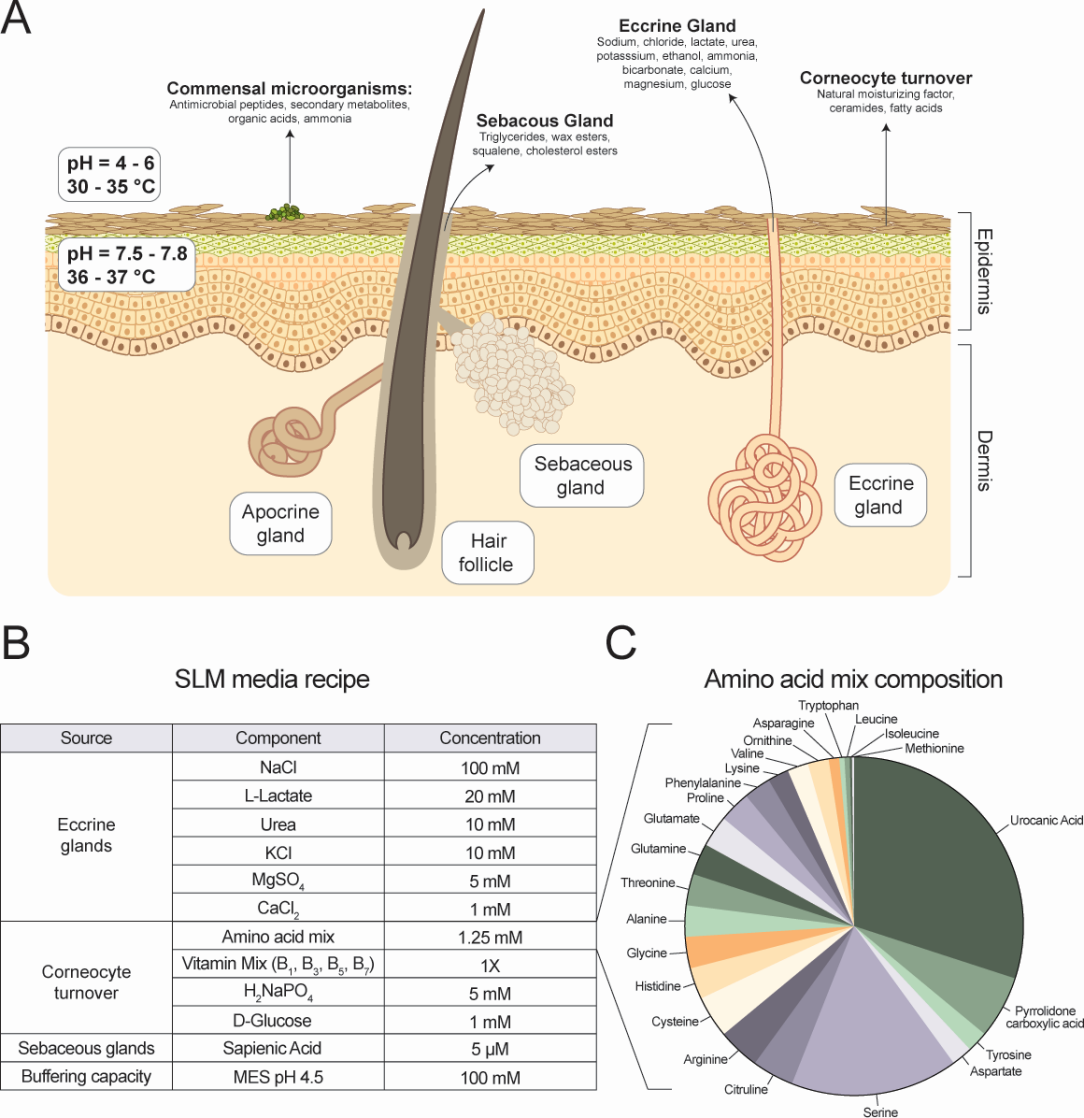
Development of the skin-like medium (SLM) recipe. (**A**) Aspects of the human skin environment that were considered in the development of the media. (**B**) Components incorporated into the media recipe and the concentrations incorporated into the media. (**C**) A pie chart demonstrating the relative concentrations of amino acids by molarity incorporated into the SLM.

The SLM formulation was informed by an extensive review of skin surface composition in the literature. Human sweat, produced by eccrine glands ubiquitously distributed on human skin, produces up to a combined 0.5–3.5 L of sweat every hour (Fig. 1A) (4, 5). We incorporated the constituents of human sweat into the media, including Na^+^, K^+^, Cl^−^, lactate, urea, Mg^2+^, and Ca^2+^ (Fig. 1B; Table S1). Additionally, we included amino acids derived from the natural moisturizing factor, a product of corneocyte turnover, in the form of water-soluble and dimethyl sulfoxide (DMSO)-soluble 40× amino acid mixes (Fig. 1B; Table S1) (22). These amino acid mixtures included the skin-specific amino acid derivatives *trans-*urocanic acid and 2-pyrrolidone-5-carboxylic acid (23, 24). Essential vitamins, sodium phosphate, and glucose were also included to provide vital nutrients for staphylococci (Fig. 1B; Table S1). Since glucose concentration on the skin surface is relatively unexplored, we added 1 mM glucose based on measurements of glucose concentration in dermal tissue and eccrine sweat gland secretions (4, 25). The human-specific fatty acid, sapienic acid, was integrated to simulate fatty acid stress of the skin while maintaining the ability to collect absorbance-based culture density measurements (Fig. 1; Table S1) (26, 27). The media were buffered to pH 4.8 using 100 mM 2-(N-morpholino)ethanesulfonic acid (MES) buffer to replicate the skin’s pH-buffered nature (28). Lastly, we set the culturing temperature to 32°C, mirroring the temperature of the anterior forearm (29). Component concentrations were determined using available quantitative data and maintaining relative proportions when absolute quantifications were not available.

To gauge *S. aureus* growth response to the SLM composition, we tested different concentrations of lactate, urea, amino acid mix, glucose, sapienic acid, and MES buffer with *S. aureus* strain LAC*, a derivative of the epidemic MRSA strain USA300_LAC that has been cured of plasmid p03, hereafter referred to as MRSA (Fig. S1) (30, 31). Lactate positively influenced the growth yield at 24 h between 10 and 30 mM; however, at 50 mM, lactate supplementation growth rate decreased, and the growth yield was lower than at 30 mM (Fig. S1A). Addition of urea produced a growth-enhancing effect that plateaued at 50 mM (Fig. S1B). The amino acid mixture proved indispensable for growth, which was unsurprising since MRSA has documented amino acid auxotrophies mentioned earlier (Fig. S1C) (15, 32). Addition of the amino acid mixture above 3.75 mM increased the lag phase and reduced the growth rate, likely due to the DMSO concentration required to keep amino acids such as *trans-*urocanic acid soluble (Fig. S1C). Surprisingly, the addition of glucose did not improve growth beyond 1 mM (Fig. S1D). Sapienic acid, which has documented inhibitory properties with staphylococci, reduced the final growth yield of MRSA starting at 20 µM and was completely inhibitory to growth at 50 µM (Fig. S1E). Finally, buffer concentration marginally influenced growth rates but did not affect final growth yields (Fig. S1F). Addition of trace metals did not improve growth, suggesting that there is sufficient trace metal contamination from the media stocks (data not shown). Since the host limits metal availability to microorganisms, supplementation of trace metals was not included in the final recipe (33).

### Growth of diverse staphylococci in SLM

**Fig 2:**
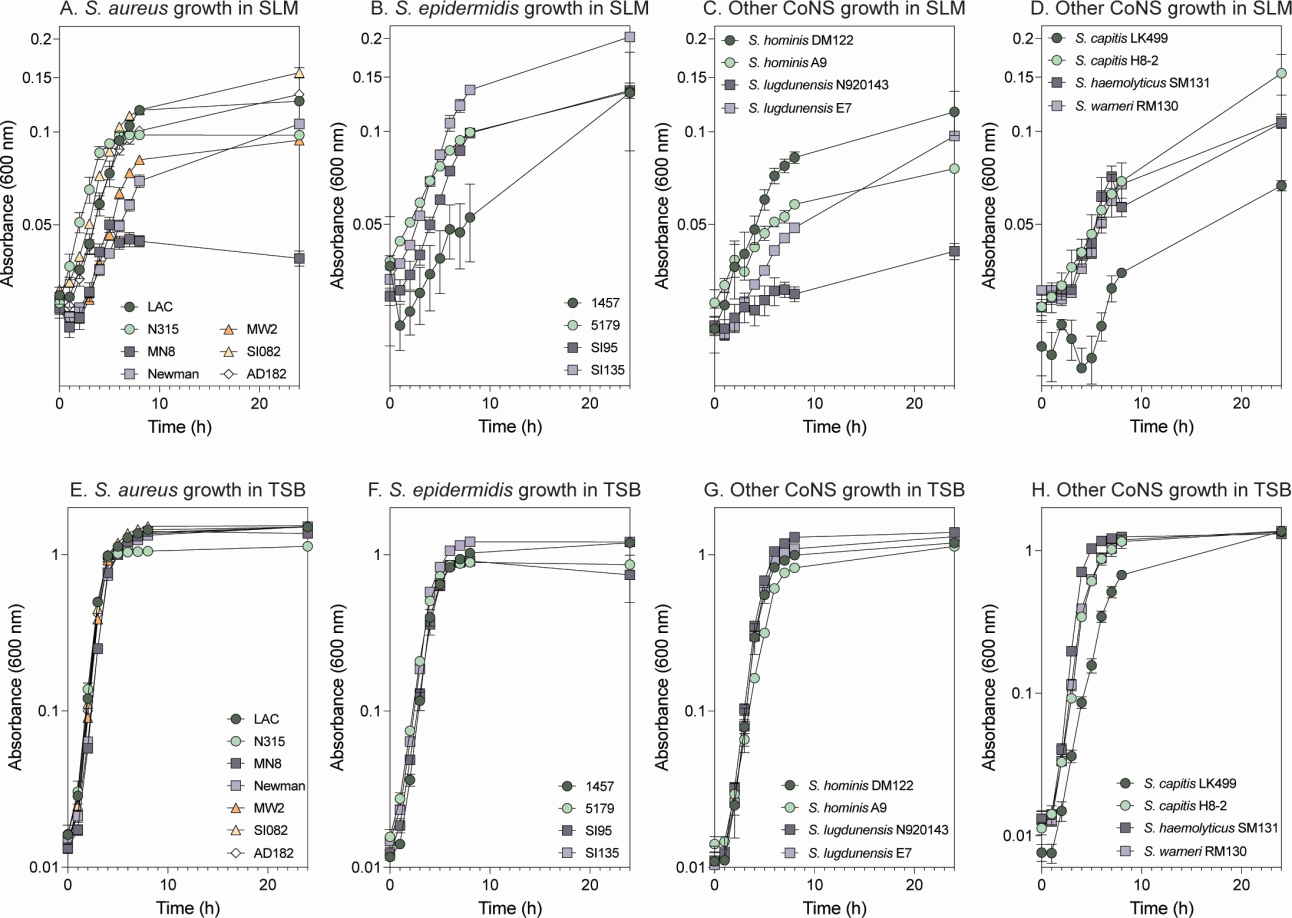
Growth of staphylococcal isolates in SLM and TSB. Growth of isolates from varied infection/colonization contexts, strain backgrounds, and staphylococcal species (Table S2) was tested in SLM (**A–D**). Heterogeneity in growth rates and growth yield after 24 h did not appear to be species dependent. The growth of these isolates was also tested in TSB as a control (**E–H**).

We next tested growth of different staphylococcal strains from infection and skin colonization contexts, including *S. aureus* and *S. epidermidis* isolates from healthy donors and atopic dermatitis patients (Table S2). All isolates demonstrated growth in SLM, with growth rates and yields varying across isolates within *S. aureus*, *S. epidermidis*, *Staphylococcus hominis*, *Staphylococcus capitis*, *Staphylococcus lugdunensis*, *Staphylococcus warneri*, and *Staphylococcus haemolyticus* species (Fig. 2A through D). Variability appeared to be isolate specific and not applicable to an entire species. As a control, the same strains were tested for growth in TSB, where these growth differences were not observed except for *S. capitis* strain LK499, which grew worse in both TSB and SLM compared to *S. capitis* strain H8-2 (Fig. 2E through H).

### Transcriptional response of MRSA in SLM

**Fig 3:**
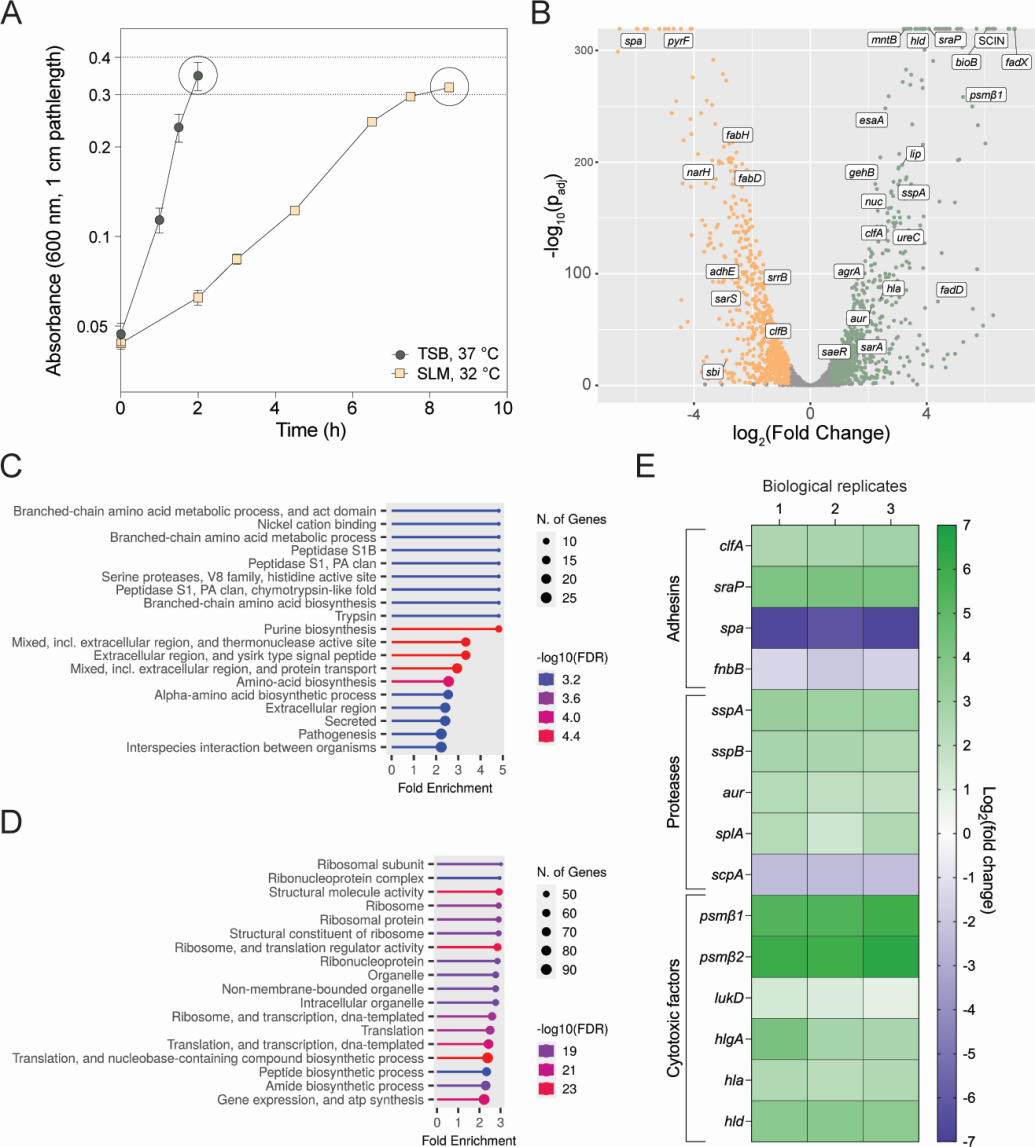
Transcriptional response of *S. aureus* to SLM. (**A**) Samples of *S. aureus* LAC* were collected at OD_600_ = 0.3 following growth in TSB at 37°C or SLM at 32°C. (**B**) A volcano plot depicting the differential expression (*x*-axis) and adjusted *P* value (*y*-axis) of each gene in the *S. aureus* LAC* genome. Upregulated genes in SLM compared to TSB are in green, and downregulated genes are in orange. A subset of genes is labeled. (**C**) Enriched pathways in the upregulated genes in SLM compared to TSB. (**D**) Enriched pathways in the downregulated genes in SLM compared to TSB. (**E**) Log_2_(fold change) of significantly differentially regulated adhesins, proteases, and cytolytic factors that play known roles in *S. aureus* colonization and virulence. Each column represents a biological replicate.

For insights into MRSA transcriptional response to SLM, we compared MRSA grown in SLM at 32°C to cultures grown in TSB at 37°C. TSB is a commonly used media for staphylococcal experiments and is composed of casein and soy peptides (20 g/L), as well as phosphate (14.3 mM), glucose (13.8 mM), and sodium chloride (85.5 mM) (34). TSB is also not buffered and has a pH of 7.2. Compared to SLM, TSB contains peptides, a higher concentration of amino acids, phosphate, and glucose, and a lower concentration of sodium chloride. Cultures were collected at the same culture density in mid to late exponential growth to normalize *agr*-dependent effects on transcription (Fig. 3A) (35). A volcano plot generated from the RNA-seq differential expression analysis demonstrated that 1,156 of the 2,629 annotated gene loci were >2-fold differentially regulated and met the adjusted *P* value cutoff (*P*_adj_ < 0.05; Fig. 3B; Table S3). Analysis of the upregulated genes using ShinyGO pathway enrichment analysis (http://bioinformatics.sdstate.edu/go/) showed that top enriched pathways in upregulated genes included purine biosynthesis (*pur* genes), branched-chain amino acid biosynthesis (*ilv* and *leu* genes), nickel cation binding (*ure* operon), and extracellular proteases (*spl* and *ssp* genes) (Fig. 3C; Table S3). Top enriched pathways in downregulated genes included functions associated with transcription and translation machinery (polymerase, ribosomal, tRNA genes, and rRNAs) and pyrimidine biosynthesis (*pyr* genes) (Table S3; Fig. 3D). The downregulated functions largely reflect the difference in growth rate between the two conditions.

Of note, many colonization and virulence factors were differentially expressed in SLM compared to TSB (Fig. 3B and E; Table S3). Upregulated adhesins included clumping factor A (*clfA*, SAUSA300_0772) and serine-rich repeat glycoprotein adhesin (*sraP* or *sasA*, SAUSA300_2589). A few adhesins were downregulated, including surface protein A (*spa*, SAUSA300_0113) and fibronectin-binding protein B (*fnbB*, SAUSA300_2440). All extracellular proteases (*splA-F*, SAUSA300_1758–1753; aureolysin [*aur*], SAUSA300_2572; and *sspA-B*, SAUSA300_0950–1), with the exception of staphopain A (*scpA*, SAUSA300_1890), were upregulated (Fig. 3E; Table S3). Several cytolytic factors and toxins were also upregulated. Most notable were the upregulation of phenol soluble modulins (PSMs) β1 and β2 (*psmβ1–2*, SAUSA300_1067–1068), which were among the most highly upregulated genes in the data set. Other cytolysins such as leukotoxins (*lukD*, SAUSA300_1768) and hemolysins (*hla*, SAUSA300_1058; *hlgABC*, SAUSA300_2365–2367; and *hld*, SAUSA300_1988) were also significantly upregulated in the RNA-seq data set (Fig. 3E; Table S3). Collectively, these changes suggest that MRSA adapts to a skin-like environment by increasing expression of virulence factors and metabolic pathways like the purine and amino acid biosynthetic pathways.

### RNA-seq comparison to MRSA transcriptional response to an *ex vivo* model

**Fig 4:**
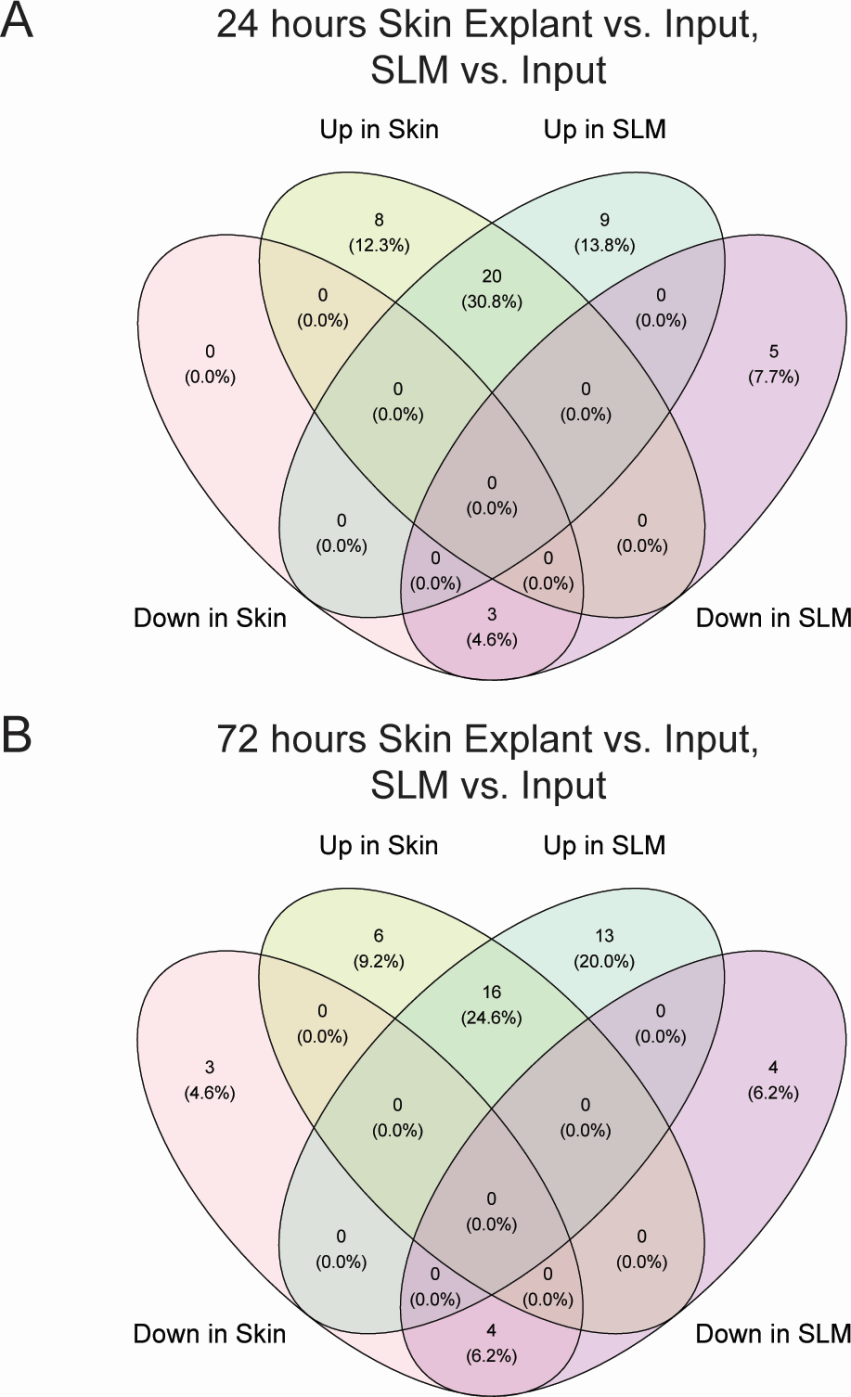
Comparison of transcriptional responses between the human skin explant data sets and SLM vs TSB RNA-seq data set. Significantly upregulated and downregulated genes from the data shown in Table S4 are shown in Venn diagrams to visualize shared upregulated and downregulated genes in (**A**) 24-h incubation on human skin explants and SLM and (**B**) 72-h incubation on human skin explants and SLM.

Although comparison of the SLM RNA-seq to an *in vivo* RNA-seq data set was not possible, we found transcriptional changes in colonization and virulence factors following growth in SLM aligned with published qRT-PCR data following MRSA application on human skin explants for 24 and 72 h (Fig. 4; Table S4). Remarkably, 35% and 30% of the compared loci in the explant study at 24 and 72 h demonstrated the same change in regulation as SLM compared to the respective TSB cultures used in the respective experiments, and none of the loci tested in the explant study conflicted with transcriptional responses in SLM and human skin explants (Fig. 4; Table S4) (36). Shared upregulated genes across all comparisons included *asp23*, *atl*, *aur*, *clfA*, *ebpS*, *esaA*, *essB*, *esxA-C*, *mntA*, SAUSA300_0883, *sraP*, *sspA*, and staphopain B (*sspB*) (Table S4). Shared downregulated genes included *clfB*, *sarS*, and *spa* (Table S4). These results suggest that MRSA grown in SLM shares some common regulatory responses with the human skin surface, particularly with loci involved in surface attachment (*clfA*, *sraP*, *clfB*, and *spa*), proteases (*aur*, *sspA*, and *sspB*), cell lysis/type VII secretion (*atl*, *esaA*, *essB*, and *esxA-C*), and transporters (*mntA*, SAUSA300_0883).

There were some genes that were upregulated at 24 h and/or 72 h in the skin explant study that were unchanged in our study. These included *csa1A*, *csa3A*, *isaA*, *isaB*, *isdA*, *isdC*, *isdG*, and *sasF*. The Csa, IsaA, and IsaB proteins have previously been identified as conserved antigens involved in immune stimulation; however, their roles in the cell have not been clearly defined (37, 38). The IsdA, IsdC, and IsdG proteins are upregulated during iron limitation, supporting that SLM is not limiting in iron despite the lack of supplementation in the media recipe (39). SasF is a sortase-anchored protein associated with fatty acid resistance (40). These differences may point to aspects of the human skin environment that have not been recapitulated in our *in vitro* model, such as the host immune system and skin lipid composition.

### Influence of pH and temperature on MRSA virulence factor expression

**Fig 5:**
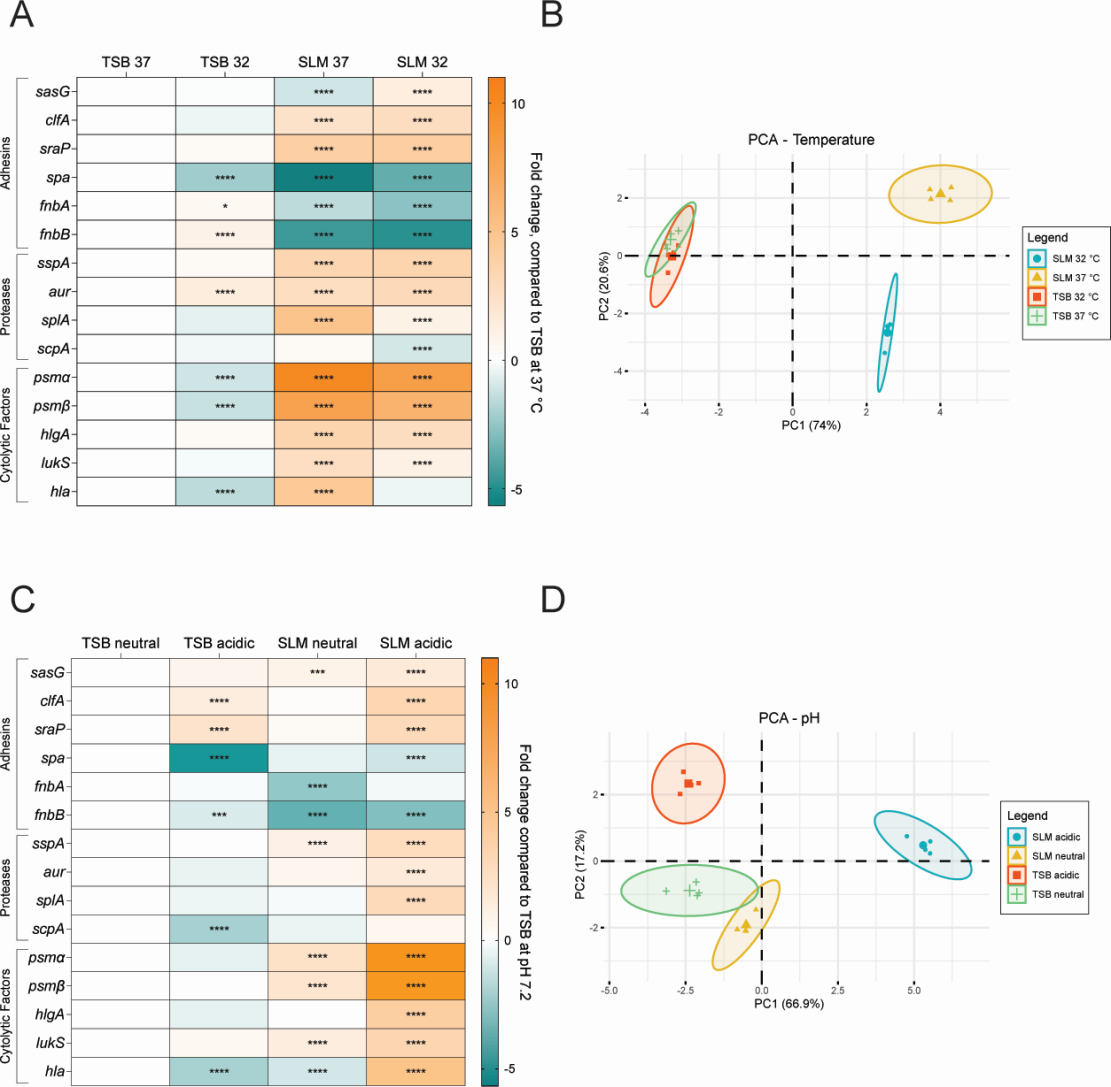
Effect of temperature and pH on virulence factor expression in TSB and SLM. (**A**) qRT-PCR in TSB and SLM at 32°C and 37°C of transcripts of genes encoding key virulence factors. (**B**) PCA plot of the fold changes shown in panel A, color-coded by condition. (**C**) qRT-PCR in TSB and SLM at pH 7.2 (neutral) and pH 4.8 (acidic) of transcripts of genes encoding key virulence factors. (**D**) PCA plot of the fold changes (see Fig. 6), color-coded by condition. Significant fold changes were determined using two-way analysis of variance and are denoted with asterisks. *P < 0.05, ***P < 0.001, ****P < 0.0001.

To validate reproducibility of the transcriptional changes in the RNA-seq and to examine the impact of temperature and pH on regulation, cultures were grown in TSB and SLM at both 32°C and 37°C, and at pH 4.8 and pH 7.2. Purified RNA was converted to cDNA, and transcription of adhesins (*sasG*, *clfA*, *sraP*, *spa*, *fnbA*, and *fnbB*), proteases (*sspA*, *aur*, *splA*, and *scpA*), and cytolytic factors (*psmα*, *psmβ*, *hlgA*, *lukS*, and *hla*) was quantified by qRT-PCR using the primers listed in Table S5. The log_2_(fold change) values were calculated by comparing the condition of interest to TSB at 37°C and neutral pH (Fig. 5). MRSA grown in SLM and TSB at both 32°C and 37°C showed some differences in transcription due to temperature (Fig. 5A). Principal component analyses (PCAs) of these data showed separation of TSB and SLM samples along PC1, suggesting that media composition and/or pH exerted a stronger influence than temperature on the regulation of these factors (Fig. 5B). Temperature also appeared to exert a stronger influence on regulation of these factors in SLM than in TSB, as noted by the separation of the SLM samples but not the TSB samples at the two temperatures (Fig. 5B). The same approach was repeated at both neutral (pH 7.2) and acidic (pH 4.8) conditions (Fig. 5C and D). These data showed more dramatic pH-dependent changes in transcription (Fig. 5C). PCA of these data showed the confidence intervals of SLM and TSB samples at pH 7.2 overlapped (Fig. 5D). When SLM and TSB media were at pH 4.8, they separated from the neutral media conditions along the first and second components, respectively (Fig. 5D). These data suggest that pH and temperature have important roles in transcription of MRSA virulence factors, and media composition influences the regulatory outcome of these signals.

### Enhanced adherence of MRSA to human corneocytes following growth in SLM

**Fig 6:**
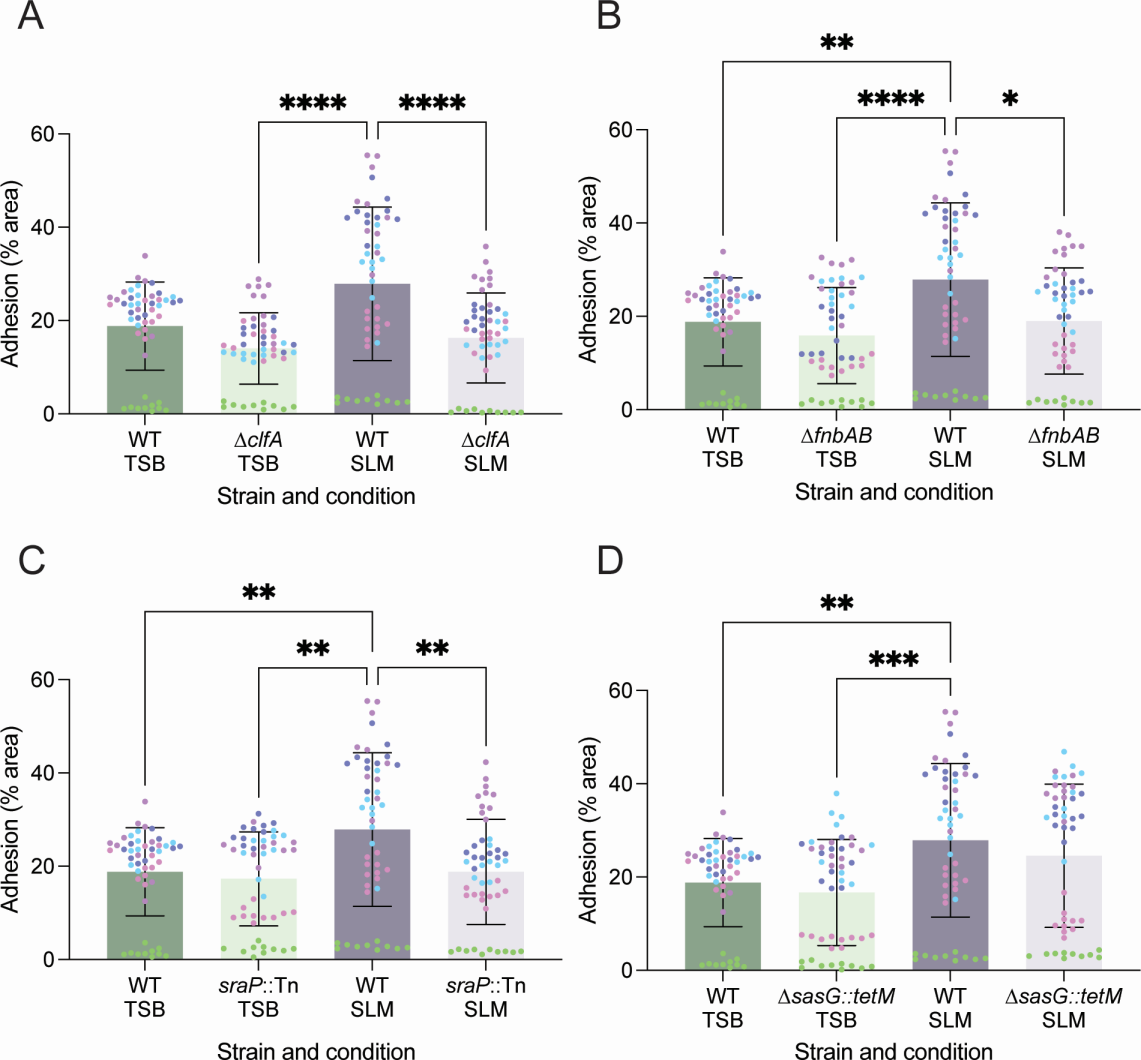
MRSA corneocyte adhesion increases following growth in SLM and is dependent on ClfA, SraP, and Fnbs. Corneocyte adhesion assays were performed with cultures grown in TSB or SLM to an OD_600_ of ~0.2. Cultures were assessed for adherence to corneocytes from five human donors (color-coded). The contributions of adhesins to corneocyte adhesion were assessed for (**A**) ClfA, (**B**) FnbA/B, (**C**) SraP, and (**D**) SasG. Cultures grown in TSB had no significant difference in adhesion. Adhesion of the wild type (WT) was significantly higher in SLM compared to TSB in three of the comparisons (**B–D**), and ClfA, FnbA/B, and SraP contributed to adhesion in the SLM-primed cells (**A–C**). SasG did not contribute to adhesion, likely due to the point mutation in *S. aureus* LAC* that prevents anchoring of SasG to the cell wall. Statistics: Kruskal-Wallis test, *P < 0.05, **P < 0.01, ***P < 0.001, ****P < 0.0001.

Previously, studies of MRSA adherence to human corneoocytes required deletion of the regulator MgrA (∆*mgrA*) or overexpression constructs to assess an adhesin’s role in corneocyte adherence (41, 42). Since genes encoding several adhesins, including *cflA* and *sraP*, were significantly upregulated in SLM in the qRT-PCR analysis, we tested whether MRSA grown in SLM adhered better to human corneocytes from the stratum corneum than when grown in TSB. With exception of the comparison in Fig. 6A, MRSA adherence to corneocytes significantly increased following growth in SLM compared to TSB (Fig. 6). The improved adherence in SLM was abrogated by mutation of *clfA*, *fnbAB*, or *sraP*, suggesting that these adhesins all contribute to MRSA adherence to human corneocytes (Fig. 6A through C). Deletion of *sasG* did not abrogate adherence (Fig. 6D). Although baseline adherence varied from donor to donor, the trends across strains were consistent (Fig. S3). Each input was dilution plated and enumerated for CFU per milliliter to ensure that inocula were consistent across strains and culture conditions (Fig. S4).

To understand how the transcriptional data shown in Fig. 5 influenced adherence, we tested adherence of *S. aureus* LAC to corneocytes when grown in SLM and TSB at different temperatures (32°C and 37°C) and pH (4.8 and 7.2). Interestingly, increased adherence to the corneocytes was observed only in SLM at pH 4.8, and temperature did not significantly affect this increased adherence (Fig. S5A). Enumeration of CFU per milliliter of the inocula confirmed that there was no difference in culture density across the conditions (Fig. S5B). These data suggest that both pH and media composition influence the adherence properties of *S. aureus*.

## DISCUSSION

Research on bacterial behavior at the human skin barrier is hindered by the limitations of available models and the institutional infrastructure required for their utilization. In this study, we generated an SLM recipe which replicates the acidity, ionic strength, buffering capacity, and known metabolic composition of an intact human skin barrier. This formulation was intentionally designed for easy assembly, requiring approximately 30 min once the necessary stocks are prepared. Moreover, the composition and pH can be adjusted for the investigation of skin niches, skin disorders, and particular metabolites of interest. Furthermore, the recipe is openly accessible for improvement as more insights into the skin’s surface environment become available. We note that another skin-like media recipe has been recently published (43). In the recipe reported herein, we have improved the incorporation of unique, skin-specific metabolites by recapitulating the measured amino acid composition of the natural moisturizing factor, which has not been included in the prior recipe.

We evaluated the growth of several staphylococcal isolates from both infection and skin colonization contexts using this media. Overall, growth yields in SLM were lower compared to nutrient-rich TSB, which was expected, given the relatively nutrient-scarce nature of the skin environment (44). All the staphylococcal isolates tested grew in SLM; however, the growth rates and yields varied considerably across isolates. The observed variability, which was not present when grown in TSB, could point to differences in the metabolic networks in these isolates, including isolate-specific auxotrophies that have not yet been identified. Exploring these metabolic differences among isolates and species in the future will contribute to a deeper understanding of the metabolic requirements of these microorganisms in their natural habitat.

**Fig 7:**
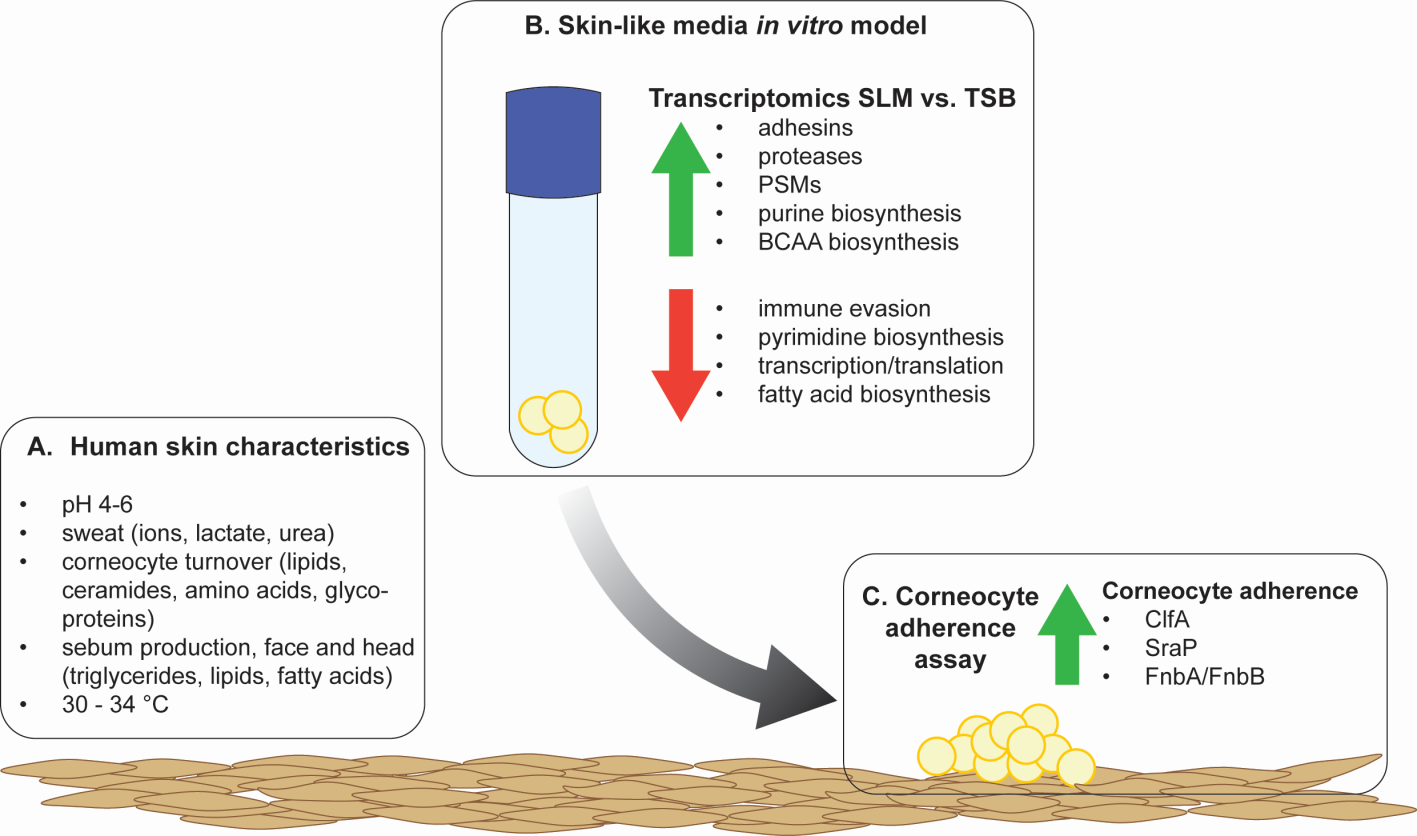
*S*. *aureus* changes its regulatory programming in skin-like conditions, priming it for improved adherence to human corneocytes. (**A**) Key human skin characteristics are listed in the box on the left. (**B**) A summary of the findings in the RNA-seq performed following *S. aureus* growth in SLM, compared to TSB. (**C**) Priming cultures in SLM resulted in improved adherence to human corneocytes that was dependent on ClfA, SraP, and Fnbps.

The MRSA strain used in these studies was isolated from a skin abscess during an outbreak investigation and is utilized as a model strain to understand MRSA pathogenesis on the skin (45). As colonization often precedes infection, we aimed to investigate MRSA responses when encountering a skin-like environment (46, 47). Analysis of the transcriptional response of MRSA following growth in SLM, compared to TSB, revealed significant changes affecting a substantial portion of the genome. There were shifts in several metabolic pathways, including the upregulation of purine and branched-chain amino acid (BCAA) biosynthesis (Fig. 7). Upregulation of purine biosynthesis was previously shown to be important for persistent infections (48), while upregulation of BCAA biosynthesis was accompanied by downregulation of the negative regulator, CodY, in SLM (49). The BCAAs are limited in abundance relative to other amino acids in the natural moisturizing factor, so it is possible that MRSA initially relies on BCAA biosynthesis rather than scavenging (22). Most downregulated pathways identified in the pathway analysis involved transcription and translation machinery; however, pyrimidine biosynthesis was identified as the most downregulated metabolic pathway in the RNA-seq data set. It is possible that this transcriptional response prevents diversion of the intermediate phosphoribosyl pyrophosphate from purine biosynthesis.

Many upregulated genes encoded for secreted and cell wall-associated factors, including adhesins, extracellular proteases, and toxins. Genes encoding the adhesins ClfA, SraP, and Ebh were upregulated in SLM. In contrast, the genes encoding IgG-binding adhesins Spa and Sbi were downregulated in SLM, as well as those encoding for adhesins ClfB and FnbB. The downregulation of FnbB was surprising, given its previously demonstrated role in corneocyte adhesion (42). In this work, we confirmed the role of the fibronectin-binding proteins in stratum corneum adherence and demonstrated roles for ClfA and SraP. The genes encoding extracellular proteases, except for *scpA*, were also upregulated. The downregulation of *scpA* in SLM may correspond to a healthy skin barrier, as qRT-PCR of mRNA collected from donors’ skin showed low expression of *scpA* in healthy controls compared to atopic dermatitis lesions and was low compared to expression of extracellular protease *sspB* in both conditions (50). Significant progress has been made in characterizing the biochemistry and substrate affinity of the Spl proteases A–F (51–53). However, with exception of SplA, the roles of the Spl proteases in colonization or infection have not been established (54). *aur* initiates the proteolytic cascade that activates V8 protease (*sspA*), which is involved in skin pruritis and in turn activates staphopain B (55–57). Additionally, several cytolytic factors and toxins were upregulated. Most notable was the upregulation of PSMs (*psmα* and *psmβ1–2*) and other cytolysins such as leukotoxins (*lukD*, SAUSA300_1768) and hemolysins (*hla*, *hlgABC*, and *hld*). PSMα and alpha toxin (Hla) both have roles in virulence and keratinocyte damage (58). Both lukS-PV and lukF-PV, which play critical roles in skin infection, had higher levels of expression in SLM compared to TSB in our RNA-seq (59). Although the increase did not meet our twofold cutoff in the RNA-seq, we observed significant upregulation of *lukS* in the qRT-PCR experiments.

The observed upregulation of the PSMs, toxins, and extracellular proteases could be explained by the upregulation of the Agr quorum sensing system, encoded by *agrBDCA* (the *agr* operon). The activation of Agr has previously been shown to be critical for MRSA-induced skin barrier damage and skin infections, and this damage can be mitigated by Agr inhibitors (60, 61). Although cultures were taken at the same density, the *agr* operon was upregulated ~2- to 3-fold. Additionally, upregulation of delta toxin, encoded by *hld*, and downregulation of *spa* and its transcriptional activator, *sarS*, are consistent with activation of Agr (62, 63). Induction of Agr could possibly be explained by de-repression from σ^B^, the transcription of which was downregulated in SLM (64). These data support the critical role of the Agr regulon in colonization and virulence on the skin (65).

In the absence of an appropriate *in vivo* comparator for SLM, we compared our RNA-sequencing data with available qRT-PCR data obtained from the same MRSA strain grown on human skin explants for 2–3 days. Strikingly, the RNA-seq data of the tested loci aligned with the transcriptional responses observed in the qRT-PCR data set, suggesting that for a subset of loci, *S. aureus* responds to the media environment similarly to how it would on the human skin surface. Most notable in this comparison was the upregulation of *clfA*. ClfA, an adhesin unique to the *Staphylococcus aureus* species, was also upregulated in another human skin explant data set (36, 66).

Both the pH and temperature of the skin environment differ from the conditions typically used for staphylococcal research *in vitro*. To understand how these factors influence regulation of MRSA colonization and virulence factors, we investigated the impact of temperature and pH on the transcription of 15 factors in both TSB and SLM. Although both parameters influenced the transcriptional response of the loci, the direction of the response was not always consistent. For instance, the expression of PSMs (*psmα* and *psmβ*) and alpha toxin (*hla*) displayed different responses to a change in pH in SLM and TSB. This discordance in response suggests that studying the effect of the skin-like environment on *S. aureus* regulation of these factors could offer insights into their roles in *S. aureus* skin colonization and virulence.

The significant shifts in regulation of the adhesins prompted investigation into *S. aureus* adherence to human corneocytes. While previous studies required genetic modifications to study *S. aureus* adherence to corneocytes (41, 42), we found that MRSA primed in SLM exhibited enhanced adherence to human corneocytes compared to priming in TSB. Additionally, adhesin phenotypes emerged in SLM-primed cultures that were not previously observed in TSB-primed cultures. In SLM-primed cultures, we identified the involvement of ClfA, the fibronectin-binding proteins (Fbps), and SraP in corneocyte adherence. While the roles of Fbps aligned with earlier research, the roles of ClfA and SraP in corneocyte adherence were novel findings (42). ClfA is unique to *S. aureus*, and the consistent upregulation of ClfA in two *ex vivo* skin explant experiments and SLM, as well as its contribution to corneocyte adherence, points to a critical role for ClfA in initial skin colonization (36, 66). Of the adhesins tested, SasG was the only one that did not contribute to corneocyte adherence. This is likely because SasG is truncated in USA300 MRSA strains such as *S. aureus* LAC* (67). It should be noted that baseline corneocyte adherence was much higher in these experiments than previously reported (41). To accommodate the growth yields in SLM, cultures were collected in TSB and SLM at a lower culture density than previously used, which may have contributed to this difference. Additionally, significant differences in adherence were observed across donors used in this study, which may point to host factors also playing a role in bacterial adherence.

It is important to note that this study has limitations, such as the limited availability of comprehensive, quantitative metabolite analyses of the skin surface to inform the recipe design and the absence of publicly available RNA-sequencing analyses of *S. aureus* on an *ex vivo* human skin model. As more information becomes available, the media recipe’s composition can be further refined, as has been observed in the iterative development of synthetic sputum media (68). Additionally, this recipe was designed for the study of staphylococci and so took into account the nutritional requirements of the genus. The study of organisms such as *Corynebacterium*, which rely on lipids as a key carbon source, would likely require addition of a synthetic sebum and additional factors as described previously (43).

The aim of this work was to develop a skin-like media recipe that facilitates cost-effective and high-throughput study of staphylococcal metabolism and physiology on the skin surface. The transcriptional response of MRSA in SLM aligns with publicly available qRT-PCR analyses of the same MRSA strain on human skin explants. Furthermore, priming MRSA strains in SLM enabled the examination of adhesin contributions to corneocyte adherence without regulatory manipulation. Collectively, this work introduces another avenue for studying staphylococcal physiology within the context of the skin environment.

## MATERIALS AND METHODS

### Strain construction

The ∆*sasG::tetM* deletion construct was inserted into pJB38 using EcoRI and SalI restriction cloning sites, comprising an fragment upstream of *sasG* amplified with primers HC246 (ATGGAATTCAATGATTTGAAAAGCAAGAGCAATA) and HC247 (CTCGAGGGTACCGCTAGCATCTCTCATTTGCATACTCCTTTTTCC), and a fragment downstream of *sasG* amplified with primers HC248 (GCTAGCGGTACCCTCGAGGCTGGATTAATGTTATTGGCACGT) and HC249 (GATGTCGACTTGATGTTATTGCAAGTAAAGGAAT). The tetracycline resistance marker, *tetM*, was amplified using primers HC3 (GTTAGCTAGCCCTAGGCAAATATGCTCTTACGTGC) and HC4 (GGCATGCTAGCGCACTAAGTTATTTTATTGAACATATATCTTAC) and was inserted into the NheI site included between the upstream and downstream fragments by the above primers. The resulting vector, pHC128, was used to generate the *sasG::tetM* deletion in LAC as described previously (69).

### Growth media

TSB (RPI cat. no. T48500) at 30 g/L was used for overnight cultures and as the reference culture condition for transcriptional analyses and phenotypic assays. For pH studies, TSB was acidified to pH 4.8 using HCl. SLM was assembled fresh using the prepared stocks and recipe outlined in Table S1A.

### Preparation of SLM

Stocks of NaCl, L-lactate, urea, KCl, H_2_NaPO_4_, MgSO_4_, CaCl_2_, and D-glucose were prepared in water and filter sterilized with a 0.22-µm MCE filter (cat. no. GSWP04700, Millipore Sigma; Table S1A). The MES stock was filter sterilized using a 0.22-µm polyethersulfone (PES) filter (cat. no. 73520–982, VWR). The 1,000× vitamin mix stock was prepared in water using the recipe outlined in Table S1B and filter sterilized with a 0.22-µm MCE filter. The 40× amino acid mix was separated into DMSO-soluble (Table S1C) and water-soluble (Table S1D) mixtures. These stocks were generated as dry mixes by weighing out the grams indicated in the “g/L” column and blending into a fine powder using a mortar and pestle. The dry mixes were stored in falcon tubes in a desiccating chamber at 4°C. The 40× DMSO-soluble amino acid mix was weighed out fresh for each experiment, solubilized in DMSO at 2.81 mg/mL, and filter-sterilized with a 0.22 µm nylon filter (cat. no. 7649-030, VWR). The 40× water-soluble amino acid mix was also weighed out fresh for each experiment, solubilized in water at 4.10 mg/mL, and filter-sterilized with a 0.22-µm MCE filter. For the qRT-PCR pH studies, 1 M 3-(N-morpholino)propanesulfonic acid (MOPS), pH 7.4 stock was prepared and replaced the 1 M MES, pH 4.5 stock in the media recipe.

### Growth conditions

Strains were grown in biological triplicate or quadruplicate in 5 mL of TSB shaking at 250 RPM in 18 mm × 150 mm glass culture tubes at 37°C for 16 h. For RNA sequencing and qRT-PCR, overnight cultures were subcultured at 1:100 (vol/vol) dilution into flasks containing TSB or SLM (20% media volume:flask volume). Cultures were shaken at 250 RPM at 32°C for SLM and 37°C in TSB to an optical density of 0.2–0.3, as measured at 600 nm with a 1-cm pathlength cuvette. For growth measurements of *S. aureus* LAC* and other staphylococci as shown in Fig. 2 and Fig. S1, cultures were subcultured at 1:100 (vol/vol) dilution in a total volume of 150 µL SLM in 96-well plates, and each biological replicate was plated in technical triplicate (Corning 3370, cat. no. 07–200-656; Fisher Scientific). Cultures were incubated at 32°C at 1,000 RPM (Stuart SI600 Incubator, Cole-Palmer). At each desired timepoint, culture density was measured by absorbance at 600 nm (Tecan Infinite200-PRO, Tecan). Technical replicates were averaged together at each timepoint for data analysis.

### RNA purification

Cultures were collected and centrifuged in falcon tubes at 3,900 *× g* for 10 min. The supernatant was discarded, and the cell pellets were quickly resuspended in 1 mL ice-cold 1× phosphate-buffered saline (PBS) and moved to a 1.7-mL microcentrifuge tube. Tubes were centrifuged at 16,000 *× g* for 30 seconds. Supernatant was aspirated, and cell pellets were flash-frozen in dry ice before storage at −80°C. RNA purification was performed within 2 weeks of freezing cell pellets as described previously (70).

### RNA-sequencing and data analysis

Purified RNA samples were submitted for rRNA depletion and mRNA sequencing (SeqCenter, Pittsburgh, PA). Samples were DNAse treated with Invitrogen DNAse (RNAse free). The library was prepared using Illumina’s Stranded Total RNA Prep Ligation with Ribo-Zero Plus kit and 10-bp Integrated DNA Technologies (IDT) for Illumina indices. Sequencing was done on a NextSeq2000, giving 2 × 51 bp reads, 12 M paired-end reads. Demultiplexing, quality control, and adapter trimming were performed with bcl-convert (v.3.9.3). The quality of resulting reads was checked using FastQC (71). Adapter removal and read trimming were performed using Trimmomatic, using the paired-end option with a minimum read length of 36 nucleotides and trimming quality of 3 (72). After trimming, read quality was again assessed with FastQC. Trimmed reads were aligned to the *S. aureus* subsp. *aureus* USA300_FPR3757 reference genome (taxonomy ID: 451515), and read counts were quantified using EDGE-pro (73). Unnormalized read counts were imported into R-Studio, and differential expression between SLM and TSB samples was quantified using DESeq2 with normalization (74). The volcano plot shown in Fig. 3B was also generated in R-Studio using the ggplot2 package.

### qRT-PCR and data analysis

Purified RNA was treated with DNase per manufacturer’s instructions (Turbo DNA-free Kit, cat. no. AM1907; Thermo Fisher Scientific). DNase-treated RNA was quantified with a Qubit 4 Fluorometer and the Qubit RNA Broad Range Assay Kit per manufacturer’s instructions (cat. no. Q10211, Thermo Fisher Scientific). One microgram of RNA was then converted to cDNA per manufacturer’s instructions and diluted to a working stock of 5 ng/µL (SuperScript IV VILO Master Mix, cat. no. 11756050; Thermo Fisher Scientific). qRT-PCR primers were designed using Primer (v.3) software (https://primer3.ut.ee/) and validated using a primer efficiency cutoff of >95%. qRT-PCR targets and associated qRT-PCR primers are listed in Table S5. Quantitative PCR was performed using PowerTrack SYBR Green Master Mix (cat. no. A46109, Thermo Fisher Scientific), and fluorescent intensity was monitored after each cycle (Bio-Rad CFX96, Bio-Rad). Reverse transcriptase-negative cDNA samples were included as a DNA contamination control. *C*_t_ values were obtained using the CFX Maestro Software (Bio-Rad), and fold changes were calculated for each locus using the 2^−∆∆Ct^ method and *gyrB* as the reference gene (75, 76).

### Corneocyte adherence assay

Donors were instructed to avoid washing with soap, hard scrubbing, and application of skincare products at the collection site in the previous 16 h, and corneocyte samples were validated by corneocyte confluency on the sticker. Bacterial strains containing the GFP-expressing vector pCM29 were used and are listed in Table S2. Strains were grown in 5 mL TSB supplemented with 10-µg/mL chloramphenicol for 16 h at 37°C, shaking at 250 RPM, and were subcultured at a 1:100 (vol/vol) dilution into flasks containing TSB or SLM (20% media volume:flask volume) supplemented with 2-µg/mL chloramphenicol. Cultures were grown at 37°C for TSB and 32°C for SLM, shaking at 250 RPM, to an OD_600_ of 0.2–0.25 as measured by cuvette (1-cm path length). Cultures were centrifuged at 3,900 × *g* for 10 min at ambient temperature, and the supernatant was aspirated. Cells were washed once in 1× PBS and normalized to ~5 × 10^7^ CFU/mL in 1× PBS, dilution plated onto TSA + chloramphenicol (10 µg/mL) plates, and incubated at 37°C overnight to enumerate CFU per milliliter. Corneocyte adherence assay and data analysis were completed as previously described (41).

## Data Availability

RNA-sequencing data were deposited in the National Institutes of Health Sequence Read Archive under BioProject PRJNA1029187.

## SUPPLEMENTAL MATERIAL

Supplemental material mbio.00453-24-s0001.docx Supplemental tables and figures.

Table S3 mbio.00453-24-s0002.csv Full table of differential expression genes from RNA-seq experiment.

ASM does not own the copyrights to Supplemental Material that may be linked to, or accessed through, an article. The authors have granted ASM a non-exclusive, world-wide license to publish the Supplemental Material files. Please contact the corresponding author directly for reuse.
