# Supplementary material for: The Staphylococcus aureus regulatory program in a human skin-like environment
Source: mBio. 2024 Mar 28;15(5):e00453-24. doi: 10.1128/mbio.00453-24 (PMC11077960; doi:10.1128/mbio.00453-24)
Supplement: Supplemental material — Supplemental tables and figures. [file mbio.00453-24-s0001.docx]

**SUPPLEMENTARY INFORMATION**

**The *Staphylococcus aureus* regulatory program in a human skin-like environment.**

Running title: *S. aureus* response to skin-like media

Flavia G. Costa^a^, Krista B. Mills^a*^, Heidi A. Crosby^a^**, Alexander R. Horswill^a,b#^

^a^Department of Immunology and Microbiology, University of Colorado Anschutz Medical Campus, Aurora, Colorado, United States of America.

^b^Department of Veterans Affairs, Eastern Colorado Healthcare System, Aurora, Colorado, United States of America.

^*^Present address: Alphabet Health (New York City, New York)

**Present address: New England Biolabs (Ipswich, Massachusetts)

^#^corresponding author

email: [alexander.horswill@cuanschutz.edu](mailto:alexander.horswill@cuanschutz.edu)

**Table S1. Detailed SLM Recipe**. (A) For each component of SLM, the stock concentration, final concentration, stock solute, and stock storage conditions are listed. (B) Preparation of the 1000X vitamin mix stock. (C) Preparation of the 40X amino acid mix stock (DMSO-soluble). (D) Preparation of the 40X amino acid mix stock (water-soluble).


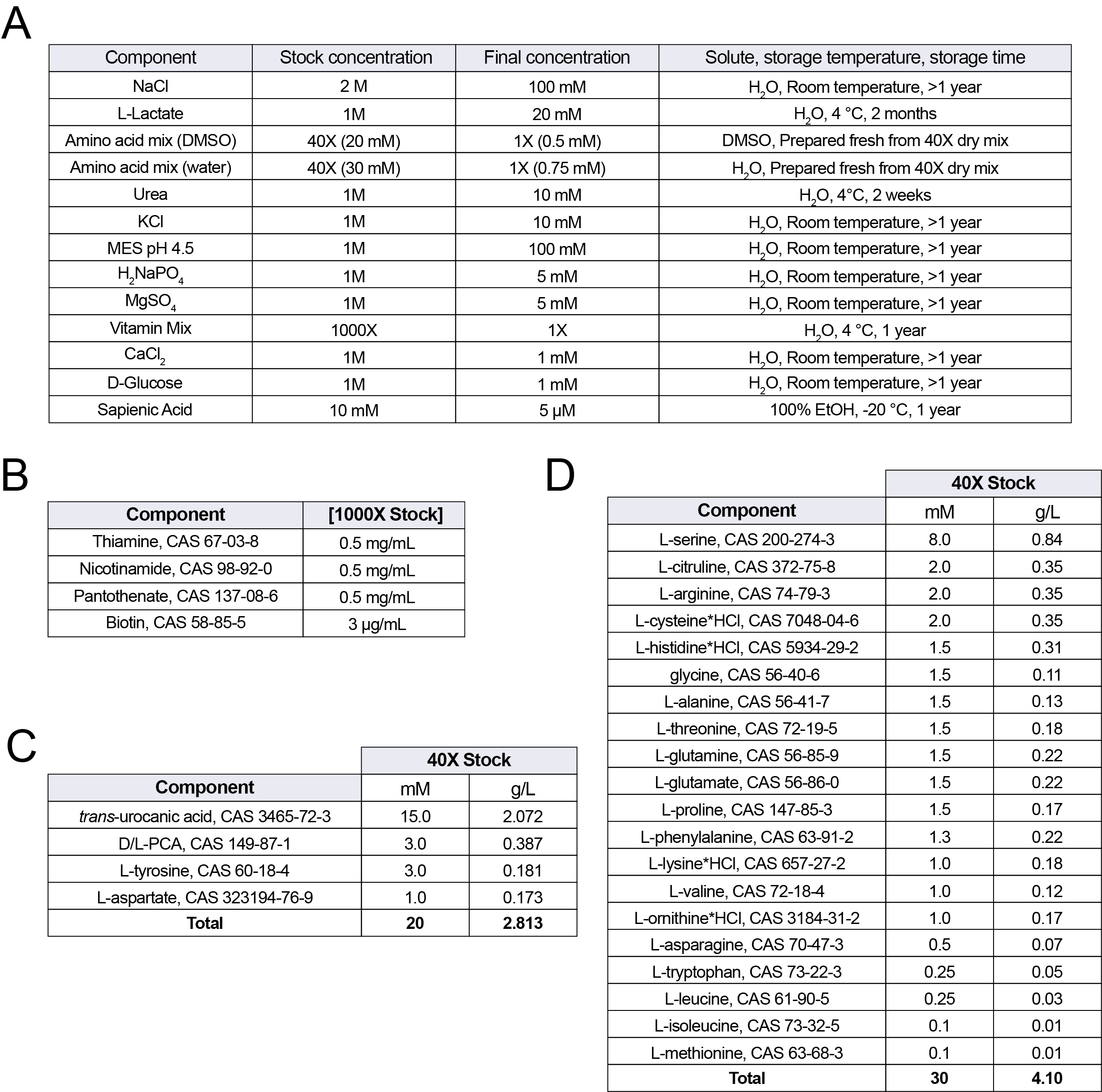


**Table S2. Strains and plasmids used in this study.**

| Skin-Like Media Growth Analysis (Figure 2) | | | | |
| --- | --- | --- | --- | --- |
| Strain No. | Species | Isolate/strain name | Isolated from | Reference |
| AH2398 | *Staphylococcus aureus* | N315 | pharyngeal smear | (2) |
| AH0579 | *Staphylococcus aureus* | MN8 | menstrual TSS | (3) |
| AH1178 | *Staphylococcus aureus* | Newman | human infection | (4) |
| AH1263 | *Staphylococcus aureus* | LAC* | skin abscess | (5) |
| AH0843 | *Staphylococcus aureus* | MW2 | septicemia | (6) |
| AH6280 | *Staphylococcus aureus* | SI082 | skin isolate | Lab biobank |
| AH5900 | *Staphylococcus aureus* | AD182 | atopic dermatitis lesion | Lab biobank |
| AH2490 | *Staphylococcus epidermidis* | 1457 | venous catheter infection | (7) |
| AH2985 | *Staphylococcus epidermidis* | 5179 | CSF-shunt infection | (8) |
| AH6072 | *Staphylococcus epidermidis* | SI95 | skin isolate | Lab biobank |
| AH6213 | *Staphylococcus epidermidis* | SI135 | skin isolate | Lab biobank |
| AH4553 | *Staphylococcus hominis* | DM122 | skin isolate | (9) |
| AH4844 | *Staphylococcus hominis* | A9 | skin isolate | (10) |
| AH3647 | *Staphylococcus lugdunensis* | N920143 | breast abscess | (11) |
| AH5012 | *Staphylococcus lugdunensis* | E7 | skin isolate | Gift from Dr. Richard Gallo |
| AH4562 | *Staphylococcus capitis* | LK499 | skin isolate | (9) |
| AH5098 | *Staphylococcus capitis* | H8-2 | skin isolate | Gift from Dr. Richard Gallo |
| AH5291 | *Staphylococcus haemolyticus* | SM131 | skin isolate | (9) |
| AH5371 | *Staphylococcus warneri* | RM130 | skin isolate | (9) |
| Corneocyte adherence assays (Figure 5) | | | | |
| Strain No. | Species | Genotype | | Reference |
| AH6274 | *S. aureus* LAC* | WT / pCM29 *cat*^+^ *sf*GFP^+^ | | (5, 16), this work |
| AH5593 | *S. aureus* LAC* | ∆*clfA* / pCM29 *cat*^+^ *sf*GFP^+^ | | (16, 17), this work |
| AH5841 | *S. aureus* LAC* | ∆*fnbAB* / pCM29 *cat*^+^ *sf*GFP^+^ | | (16, 18), this work |
| AH6272 | *S. aureus* LAC* | *sraP*::Tn / pCM29 *cat*^+^ *sf*GFP^+^ | | (16, 19), this work |
| AH6273 | *S. aureus* LAC* | ∆*sasG::tetM* / pCM29 *cat*^+^ *sf*GFP^+^ | | this work |

**Table S3. Differential expression analysis of *S. aureus* LAC following growth in SLM, compared to TSB.** Collected cultures were processed for RNA purification, and further processed at SeqCenter for rRNA depletion and RNA-sequencing. Raw reads were trimmed with Trimmomatic and assessed for quality with FastQC. Reads were aligned to the *S. aureus* FPR3757 genome with EDGE-pro, and differential expression of read counts were analyzed with DESeq2 in RStudio. Genes with p_adj_ > 0.05 were excluded from list.

**Table in separate Excel file**

**Table S4. Comparison of SLM vs TSB RNA-seq to qRT-PCR data from a human skin explant.** Data collected in a previous qRT-PCR study of *S. aureus* colonization of human skin explants at 24- and 72-hours vs input,(1) was compared to the same subset of loci in the SLM vs TSB RNA-seq dataset. Significance was assessed by >2 foldchange and p_value_ <0.05 for qRT-PCR data and p_adj_ < 0.05 for the RNA-seq data. UP = upregulated in (condition) vs input, NS = below cutoffs, DN = downregulated in (condition) vs input.

| Gene Name | Gene Loci | 24h Skin v Input qRT-PCR | 72h Skin v Input qRT-PCR | SLM v TSB RNA-seq |
| --- | --- | --- | --- | --- |
| *adsA* | SAUSA300_0025 | NS | NS | NS |
| *agrA* | SAUSA300_1992 | NS | NS | UP |
| *ahpC* | SAUSA300_0380 | NS | NS | NS |
| *asp23* | SAUSA300_2142 | UP | UP | UP |
| *atl* | SAUSA300_0955 | UP | UP | UP |
| *aur* | SAUSA300_2572 | UP | UP | UP |
| *chp* | SAUSA300_1920 | NS | DN | NS |
| *clfA* | SAUSA300_0772 | UP | UP | UP |
| *clfB* | SAUSA300_2565 | DN | DN | DN |
| *coa* | SAUSA300_0224 | NS | NS | DN |
| *csa1A* | SAUSA300_0100 | UP | UP | NS |
| *csa2A* | SAUSA300_0205 | NS | NS | NS |
| *csa3A* | SAUSA300_0410 | UP | UP | NS |
| *csa4A* | SAUSA300_2424 | NS | NS | NS |
| *eap* | SAUSA300_1917 | NS | NS | NS |
| *ebpS* | SAUSA300_1370 | UP | UP | UP |
| *esaA* | SAUSA300_0279 | UP | UP | UP |
| *esaB* | SAUSA300_0281 | NS | UP | UP |
| *essA* | SAUSA300_0280 | NS | NS | UP |
| *essB* | SAUSA300_0282 | UP | UP | UP |
| *essC* | SAUSA300_0283 | UP | NS | UP |
| *esxA* | SAUSA300_0278 | UP | UP | UP |
| *esxB* | SAUSA300_0285 | UP | UP | UP |
| *esxC* | SAUSA300_0284 | UP | UP | UP |
| *eta* | SAUSA300_1065 | NS | NS | NS |
| *fbp* | SAUSA300_1101 | NS | NS | NS |
| *fhuD2* | SAUSA300_2235 | NS | NS | DN |
| *flr* | SAUSA300_1053 | NS | NS | UP |
| *fnbA* | SAUSA300_2441 | NS | NS | NS |
| *gehB* | SAUSA300_0320 | UP | NS | UP |
| *hla* | SAUSA300_1058 | UP | NS | UP |
| *hlb-2* | SAUSA300_1973 | NS | NS | DN |
| *hlgB* | SAUSA300_2367 | UP | NS | UP |
| *icaB* | SAUSA300_2601 | NS | NS | NS |
| *isaA* | SAUSA300_2506 | UP | UP | NS |
| *isaB* | SAUSA300_2573 | UP | UP | NS |
| *isdA* | SAUSA300_1029 | UP | NS | NS |
| *isdB* | SAUSA300_1028 | NS | NS | NS |
| *isdC* | SAUSA300_1030 | UP | UP | NS |
| *isdG* | SAUSA300_1035 | UP | NS | NS |
| *isdH* | SAUSA300_1677 | NS | NS | UP |
| *ltaA* | SAUSA300_0917 | NS | NS | NS |
| *lukE* | SAUSA300_1769 | NS | NS | NS |
| *lukH* | SAUSA300_1975 | UP | NS | UP |
| *mgrA* | SAUSA300_0672 | NS | DN | NS |
| *mntA* | SAUSA300_0620 | UP | UP | UP |
| *nuc2* | SAUSA300_1222 | NS | NS | DN |
| *saeP* | SAUSA300_0693 | NS | NS | UP |
| SAUSA300_0883 | SAUSA300_0883 | UP | UP | UP |
| SAUSA300_1217 | SAUSA300_1217 | NS | NS | UP |
| *sarA* | SAUSA300_0605 | NS | NS | UP |
| *sarS* | SAUSA300_0114 | DN | DN | DN |
| *sarZ* | SAUSA300_2331 | NS | DN | NS |
| *sraP* | SAUSA300_2589 | UP | UP | UP |
| *fmtB* | SAUSA300_2110 | NS | NS | NS |
| *sasC* | SAUSA300_1702 | NS | NS | NS |
| *sasD* | SAUSA300_0136 | NS | NS | NS |
| *sasF* | SAUSA300_2581 | UP | UP | NS |
| *sasG* | SAUSA300_2436 | NS | NS | NS |
| *sbi* | SAUSA300_2364 | NS | DN | DN |
| *scpA* | SAUSA300_1445 | NS | NS | NS |
| *sdrC* | SAUSA300_0546 | NS | NS | UP |
| *spa* | SAUSA300_0113 | DN | DN | DN |
| *sspA* | SAUSA300_0951 | UP | UP | UP |
| *sspB* | SAUSA300_0950 | UP | UP | UP |

**Table S5. Primers designed for qRT-PCR.** qRT-PCR primers for each loci were designed with Primer 3 and validated for primer efficiency >95%.

| **Gene name** | **Locus** | **Forward primer** | **Reverse primer** |
| --- | --- | --- | --- |
| *gyrB* | SAUSA300_0005 | AATTATTCTCAGAGCACTTTGAAC | TACATCTAACGCTGATTTACGA |
| *sasG* | SAUSA300_2436 | TGTATTTGGGAACTCAACAAGA | GCAGTGTCTTTGTTTGTTACTT |
| *clfA* | SAUSA300_0772 | CAGCACAACAGGAAACGACA | GATTGAGTTGTTGCCGGTGT |
| *sraP* | SAUSA300_2589 | GCTTCTGATGCACCATTAAC | CTGTTGATGTTGATGCTTCG |
| *spa* | SAUSA300_0113 | ATTCGTAAACTAGGTGTAGGTATT | TTACGCCACCAGATATAAGTAAT |
| *fnbA* | SAUSA300_2441 | GCACAACCAGCAAATATAGAAA | CTTGAGATTGTGTTGTTTCCTT |
| *fnbB* | SAUSA300_2440 | ATAACGCAGAAAGAGTAACCTT | AGTGTTGAGATACCATGAGTTT |
| *sspA* | SAUSA300_0951 | ACCAGCAACAATGAGTAATAATG | AAATCATATTGCATAGCTTCGC |
| *aur* | SAUSA300_2572 | CCGAGTGTTGATGGTGTTCA | TCTTCGCATCAGTATCCCCA |
| *splA* | SAUSA300_1758 | ATGTCAAAGAAATTACCGATGC | GCGATATGTTTGTTAGTTACGAT |
| *scpA* | SAUSA300_1445 | CCCCTATTGCAAACGCTGAG | TGCGCTAAATTACGTACGTCA |
| *psmα3-4* | SAUSA300_0424.3-.4 | GGCCATTCACATGGAATTCGT | GCCATCGTTTTGTCCTCCTG |
| *psmβ1* | SAUSA300_1068 | TGATAGTGTGAAATTAGGCA | AGAATCCAAATAATTTACCTAGTA |
| *hlgA* | SAUSA300_2365 | AGAACACAAGACATTACTAGCA | CTAATGAAGCCTTGCATCTTAA |
| *lukS-*PV | SAUSA300_1382 | ACATTAGGTTATAACATAGGTGGT | TTTGACGTTCTACTTCACTGAT |
| *hla* | SAUSA300_1058 | AGTTATTAGAACGAAAGGTACCA | ACTTCATTATCAGGTAGTTGCA |


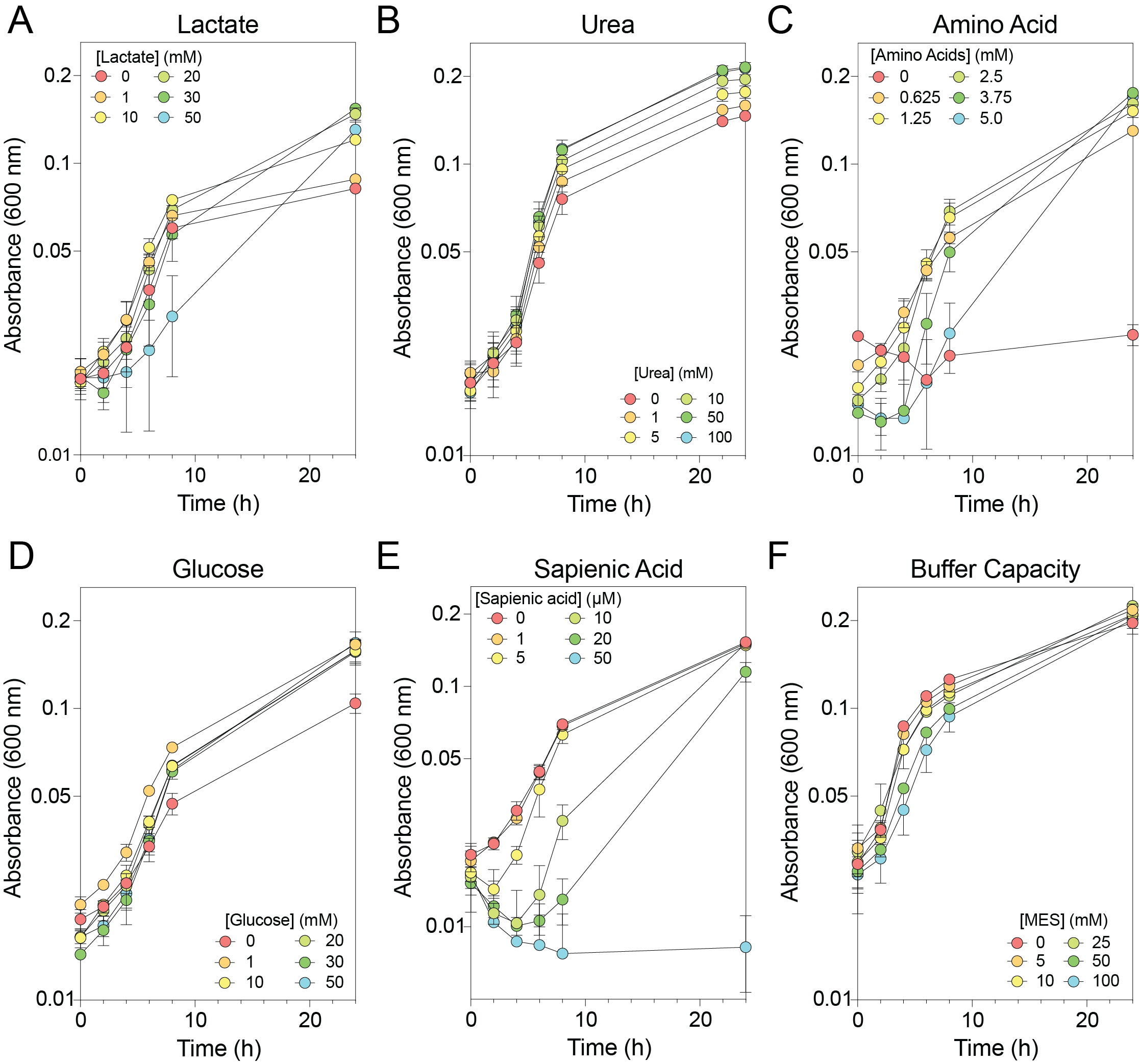


**Figure S1. Growth of *S. aureus* LAC in response to various concentrations of SLM components.** *S. aureus* LAC was grown in SLM where all component concentrations were kept consistent with exception of the component listed. *S. aureus* growth in varying concentrations of A) L-lactate, B) urea, C) amino acid mixture, D) glucose, E) sapienic acid, and F) MES buffer were tested.

**
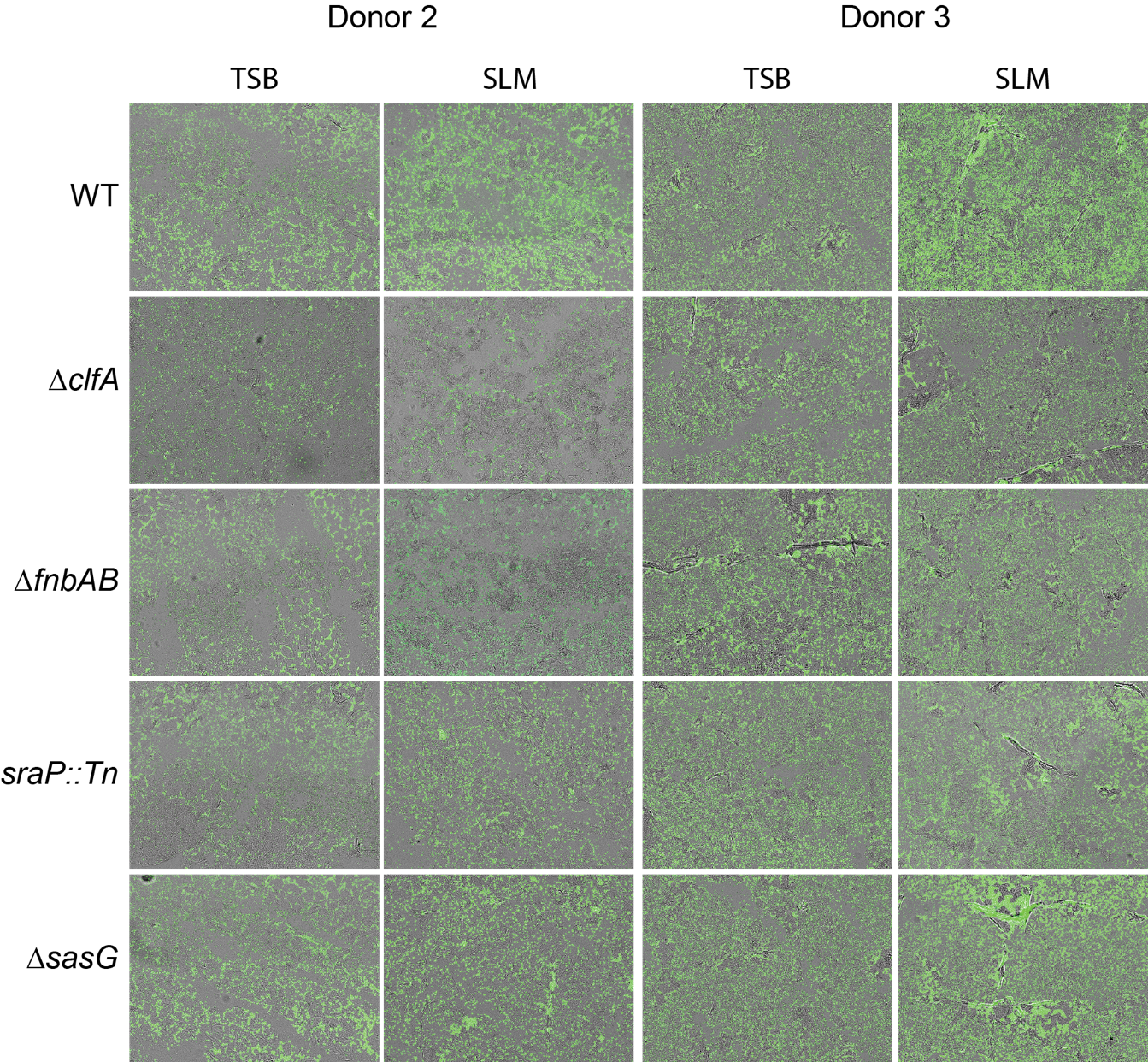
**

**Figure S2. Representative images from corneocyte adherence assays.** Examples of microscopy images obtained from corneocyte adherence assays from two of the five donors.

**
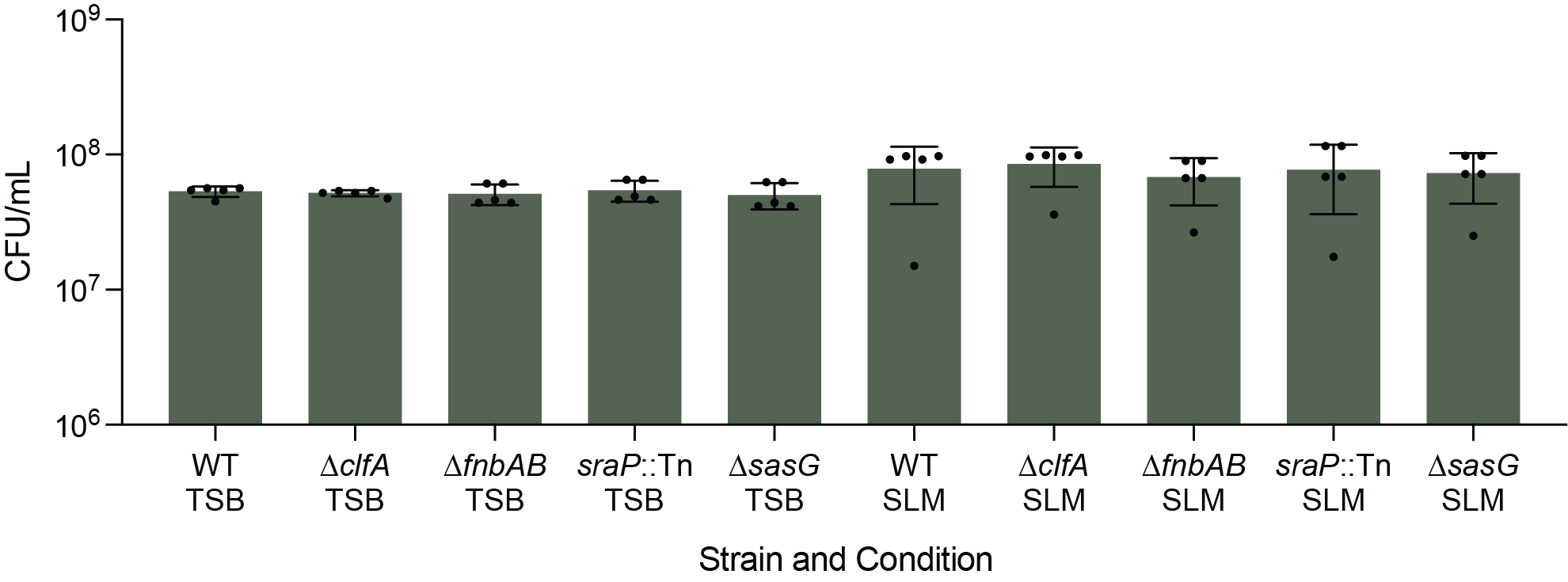
**

**Figure S3. Dilution plating of corneocyte assay input.** For each corneocyte assay experiment, washed and normalized cultures were dilution plated on TSA + chloramphenicol (10 μg/mL) plates and enumerated for colony-forming units (CFUs). No significance found with one-way ANOVA.

**
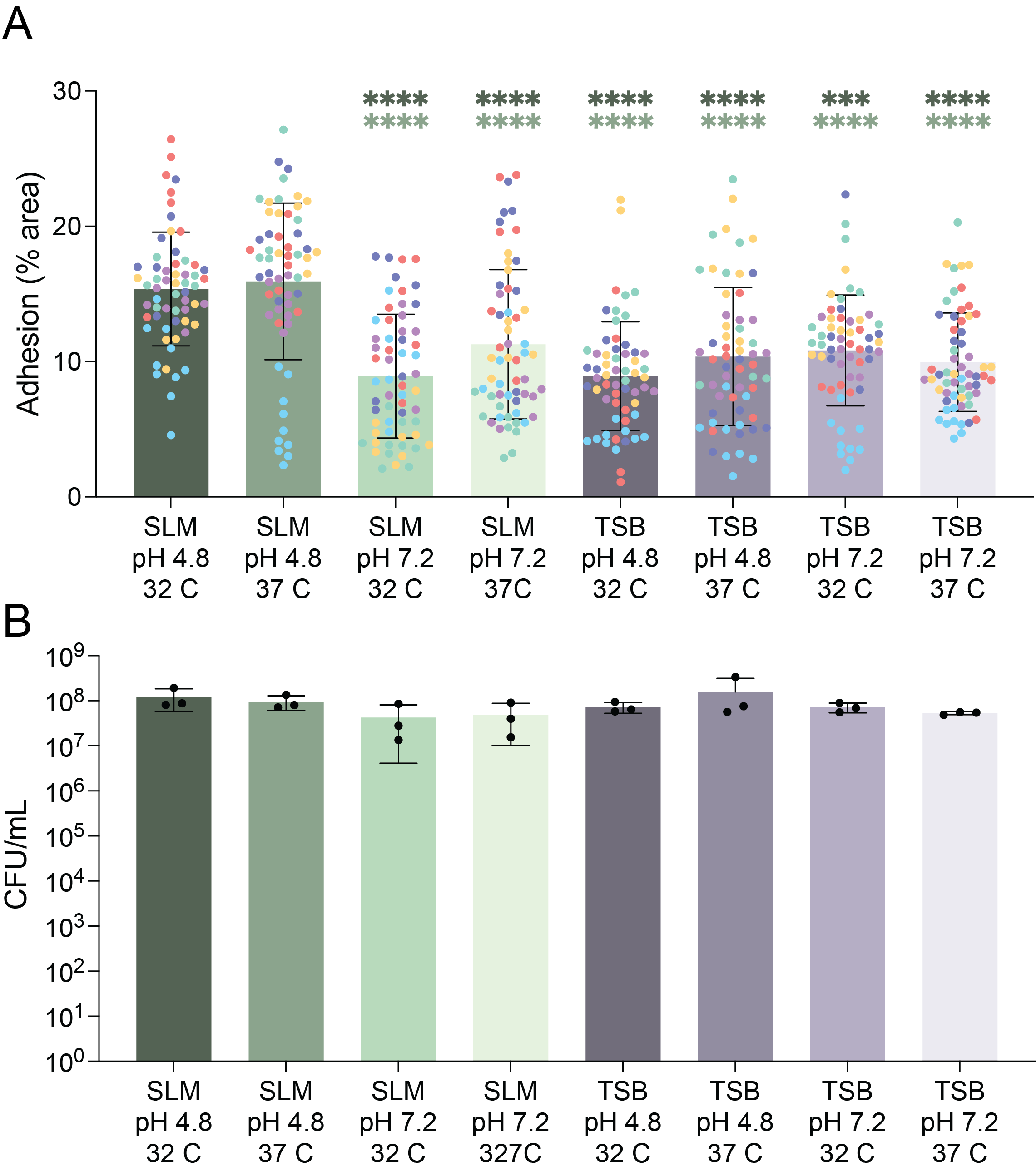
**

**Figure S4. Effect of media, pH, and temperature on *S. aureus* corneocyte adhesion.** A) Corneocyte adhesion assays were performed with *S. aureus* LAC grown in TSB or SLM, at pH 4.8 or 7.2, and 32 °C or 37 °C, to OD_600_ ~0.2. Cultures were assessed for adherence to corneocytes from six human donors (color-coded). Using the Kruskal-Wallis test, p<0.05, increased adherence was observed in SLM at a pH of 4.8 at both temperatures compared to all other conditions, no significance was found across other comparisons. B) For each corneocyte assay experiment, washed and normalized cultures were dilution plated on TSA + chloramphenicol (10 µg/mL) plates and enumerated for colony-forming units (CFUs). No significance found with one-way ANOVA.

**SUPPLEMENTAL REFERENCES**

1. Cruz AR, van Strijp JAG, Bagnoli F, Manetti AGO. 2021. Virulence Gene Expression of *Staphylococcus aureus* in Human Skin. Front Microbiol 12:692023.

2. Kuroda M, Ohta T, Uchiyama I, Baba T, Yuzawa H, Kobayashi I, Cui L, Oguchi A, Aoki K, Nagai Y, Lian J, Ito T, Kanamori M, Matsumaru H, Maruyama A, Murakami H, Hosoyama A, Mizutani-Ui Y, Takahashi NK, Sawano T, Inoue R, Kaito C, Sekimizu K, Hirakawa H, Kuhara S, Goto S, Yabuzaki J, Kanehisa M, Yamashita A, Oshima K, Furuya K, Yoshino C, Shiba T, Hattori M, Ogasawara N, Hayashi H, Hiramatsu K. 2001. Whole genome sequencing of methicillin-resistant *Staphylococcus aureus*. Lancet 357:1225-1240.

3. Recsei P, Kreiswirth B, O'Reilly M, Schlievert P, Gruss A, Novick RP. 1986. Regulation of exoprotein gene expression in *Staphylococcus aureus* by *agr*. Mol Gen Genet 202:58-61.

4. Duthie ES, Lorenz LL. 1952. Staphylococcal coagulase; mode of action and antigenicity. J Gen Microbiol 6:95-107.

5. Kennedy AD, Otto M, Braughton KR, Whitney AR, Chen L, Mathema B, Mediavilla JR, Byrne KA, Parkins LD, Tenover FC, Kreiswirth BN, Musser JM, DeLeo FR. 2008. Epidemic community-associated methicillin-resistant *Staphylococcus aureus*: recent clonal expansion and diversification. Proc Natl Acad Sci USA 105:1327-1332.

6. Anonymous. 1999. From the Centers for Disease Control and Prevention. Four pediatric deaths from community-acquired methicillin-resistant Staphylococcus aureus--Minnesota and North Dakota, 1997-1999. JAMA 282:1123-5.

7. Mack D, Siemssen N, Laufs R. 1992. Parallel induction by glucose of adherence and a polysaccharide antigen specific for plastic-adherent *Staphylococcus epidermidis*: evidence for functional relation to intercellular adhesion. Infect Immun 60:2048-2057.

8. Rohde H, Knobloch JK, Horstkotte MA, Mack D. 2001. Correlation of biofilm expression types of *Staphylococcus epidermidis* with polysaccharide intercellular adhesin synthesis: evidence for involvement of icaADBC genotype-independent factors. Med Microbiol Immunol 190:105-112.

9. Schleifer KH, Kloos WE. 1975. Isolation and Characterization of Staphylococci from Human Skin I. Amended Descriptions of *Staphylococcus epidermidis* and *Staphylococcus saprophyticus* and Descriptions of Three New Species: *Staphylococcus cohnii*, *Staphylococcus haemolyticus*, and *Staphylococcus xylosus*. International Journal of Systematic and Evolutionary Microbiology 25:50-61.

10. Nakatsuji T, Chen TH, Narala S, Chun KA, Two AM, Yun T, Shafiq F, Kotol PF, Bouslimani A, Melnik AV, Latif H, Kim JN, Lockhart A, Artis K, David G, Taylor P, Streib J, Dorrestein PC, Grier A, Gill SR, Zengler K, Hata TR, Leung DY, Gallo RL. 2017. Antimicrobials from human skin commensal bacteria protect against *Staphylococcus aureus* and are deficient in atopic dermatitis. Sci Transl Med 9:1-11.

11. Heilbronner S, Holden MT, van Tonder A, Geoghegan JA, Foster TJ, Parkhill J, Bentley SD. 2011. Genome sequence of *Staphylococcus lugdunensis* N920143 allows identification of putative colonization and virulence factors. FEMS Microbiol Lett 322:60-67.

12. Riegel P, de Briel D, Prevost G, Jehl F, Monteil H, Minck R. 1993. Taxonomic study of Corynebacterium Group ANF-1 strains: Proposal of Corynebacterium afermentans sp. nov. containing the subspecies C. afermentans subsp. afermentans subsp. nov. and C. afermentans subsp. lipophilum subsp. nov. Int J Syst Bacteriol 43:287-92.

13. Kocur M, Pačova Z, Martinec T. 1972. Taxonomic Status of *Micrococcus luteus* (Schroeter 1872) Cohn 1872, and Designation of the Neotype Strain. International Journal of Systematic and Evolutionary Microbiology 22:218-223.

14. Stalhammar-Carlemalm M, Areschoug T, Larsson C, Lindahl G. 1999. The R28 protein of *Streptococcus pyogene*s is related to several group B streptococcal surface proteins, confers protective immunity and promotes binding to human epithelial cells. Mol Microbiol 33:208-219.

15. Sumby P, Porcella SF, Madrigal AG, Barbian KD, Virtaneva K, Ricklefs SM, Sturdevant DE, Graham MR, Vuopio-Varkila J, Hoe NP, Musser JM. 2005. Evolutionary origin and emergence of a highly successful clone of serotype M1 group a Streptococcus involved multiple horizontal gene transfer events. J Infect Dis 192:771-782.

16. Pang YY, Schwartz J, Thoendel M, Ackermann LW, Horswill AR, Nauseef WM. 2010. agr-Dependent interactions of *Staphylococcus aureus* USA300 with human polymorphonuclear neutrophils. J Innate Immun 2:546-559.

17. Kwiecinski JM, Crosby HA, Valotteau C, Hippensteel JA, Nayak MK, Chauhan AK, Schmidt EP, Dufrene YF, Horswill AR. 2019. *Staphylococcus aureus* adhesion in endovascular infections is controlled by the ArlRS-MgrA signaling cascade. PLoS Pathog 15:e1007800.

18. Deng L, Schilcher K, Burcham LR, Kwiecinski JM, Johnson PM, Head SR, Heinrichs DE, Horswill AR, Doran KS. 2019. Identification of Key Determinants of Staphylococcus aureus Vaginal Colonization. mBio 10.

19. Crosby HA, Schlievert PM, Merriman JA, King JM, Salgado-Pabon W, Horswill AR. 2016. The *Staphylococcus aureus* Global Regulator MgrA Modulates Clumping and Virulence by Controlling Surface Protein Expression. PLoS Pathog 12:e1005604.
